# scPRAM accurately predicts single-cell gene expression perturbation response based on attention mechanism

**DOI:** 10.1093/bioinformatics/btae265

**Published:** 2024-04-15

**Authors:** Qun Jiang, Shengquan Chen, Xiaoyang Chen, Rui Jiang

**Affiliations:** MOE Key Laboratory of Bioinformatics and Bioinformatics Division of BNRIST, Department of Automation, Tsinghua University, Beijing 100084, China; School of Mathematical Sciences and LPMC, Nankai University, Tianjin 300071, China; MOE Key Laboratory of Bioinformatics and Bioinformatics Division of BNRIST, Department of Automation, Tsinghua University, Beijing 100084, China; MOE Key Laboratory of Bioinformatics and Bioinformatics Division of BNRIST, Department of Automation, Tsinghua University, Beijing 100084, China

## Abstract

**Motivation:**

With the rapid advancement of single-cell sequencing technology, it becomes gradually possible to delve into the cellular responses to various external perturbations at the gene expression level. However, obtaining perturbed samples in certain scenarios may be considerably challenging, and the substantial costs associated with sequencing also curtail the feasibility of large-scale experimentation. A repertoire of methodologies has been employed for forecasting perturbative responses in single-cell gene expression. However, existing methods primarily focus on the average response of a specific cell type to perturbation, overlooking the single-cell specificity of perturbation responses and a more comprehensive prediction of the entire perturbation response distribution.

**Results:**

Here, we present scPRAM, a method for predicting perturbation responses in single-cell gene expression based on attention mechanisms. Leveraging variational autoencoders and optimal transport, scPRAM aligns cell states before and after perturbation, followed by accurate prediction of gene expression responses to perturbations for unseen cell types through attention mechanisms. Experiments on multiple real perturbation datasets involving drug treatments and bacterial infections demonstrate that scPRAM attains heightened accuracy in perturbation prediction across cell types, species, and individuals, surpassing existing methodologies. Furthermore, scPRAM demonstrates outstanding capability in identifying differentially expressed genes under perturbation, capturing heterogeneity in perturbation responses across species, and maintaining stability in the presence of data noise and sample size variations.

**Availability and implementation:**

https://github.com/jiang-q19/scPRAM and https://doi.org/10.5281/zenodo.10935038.

## 1 Introduction

In the presence of external perturbations such as drug treatments, infections, and genetic editing, cells undergo corresponding changes in various characteristics (Peidli *et al.* 2022). Investigating these changes at the level of single-cell gene expression contributes to a deeper understanding of the impacts of perturbations, thereby providing insights for pharmacological research, clinical therapy, and medical diagnosis (Burkhardt *et al.* 2021, Wills *et al.* 2013). For example, utilizing perturbation data from previous cases to predict the response of each patient to the same drug can provide a reference for personalized treatment (Ding *et al.* 2020, Wang *et al.* 2022). The rapid evolution of single-cell RNA sequencing (scRNA-seq) techniques in recent years has made it feasible to study perturbation responses at a single-cell resolution (Metzker 2010, Gao *et al.* 2023). However, obtaining perturbed tissue samples can be challenging, and the substantial costs associated with sequencing hinder the feasibility of large-scale experimentation (Dal Molin and Di Camillo 2019). Therefore, employing bioinformatics methods to predict cellular gene expression responses to perturbations becomes essential.

In recent years, researchers have sought to apply machine learning methods to analyze perturbation responses in single-cell genomics. Ji *et al.* (2021) provided a comprehensive review of employing machine learning for perturbation modeling, recognizing perturbation response prediction as a crucial research objective within perturbation modeling. They highlighted nonlinear distribution modeling as a common approach for predicting perturbation responses. Representative methods of this approach include scGen (Lotfollahi *et al.* 2019), trVAE (Lotfollahi *et al.* 2020), and scVIDR (Kana *et al.* 2023). scGen employs a variational autoencoder (VAE) (Kingma and Welling 2014) to capture latent encodings and employs a vector algorithm to calculate the perturbation vector. However, it disregards the heterogeneity of perturbation responses across different cell types, merely averaging perturbation vectors for each type. scVIDR considers cell type heterogeneity and employs a linear regression model to predict perturbation vectors for unseen cells. Nevertheless, scVIDR overlooks the heterogeneity even among cells of the same type and the limited sample size of the linear regression model may lead to insufficient training. trVAE employs a conditional VAE (Sohn *et al.* 2015) and maximum mean discrepancy (MMD) (Borgwardt *et al.* 2006) for style transfer between pre-perturbation and post-perturbation states. This approach is computationally intensive during MMD calculation and struggles to retain original biological information effectively. Beyond the VAE-based methods, Wei *et al.* (2022) proposed scPreGAN, utilizing generative adversarial networks (GANs) (Goodfellow *et al.* 2020) for predicting perturbation responses. However, GAN training instability and the resultant predictions neglect the biological information of target cells. CellOT (Bunne *et al.* 2023) utilizes optimal transport based on input convex neural networks (Makkuva *et al.* 2020) to directly map cells from their undisturbed states to perturbed states. However, the performance of direct mapping significantly declines when confronted with new types of data out of the training samples.

To address the aforementioned limitations, we introduce scPRAM, a novel approach for predicting perturbation responses in single-cell gene expression based on an attention mechanism (Vaswani *et al.* 2017). scPRAM leverages a VAE to encode the training set into a latent space, followed by optimal transport based on Sinkhorn algorithm (Villani 2009) to pair unpaired cells. Subsequently, an attention mechanism is employed to compute perturbation vectors for test cells. This method takes into full consideration the heterogeneity of perturbation responses in the gene expression of individual cells, as the perturbation load for each test cell is inherently different. We have demonstrated across multiple datasets and various metrics that scPRAM predicts single-cell gene expression perturbations much better than existing methods. Besides, scPRAM can predict heterogeneous perturbation responses for new cell types across cell types, species, and individuals, and it exhibits excellent robustness. Furthermore, scPRAM is capable of predicting a greater number of differentially expressed genes (DEGs) for downstream enrichment analysis, providing insights into understanding perturbation-related biological responses.

## 2 Materials and methods

### 2.1 Overview of the scPRAM model

**Figure 1.**
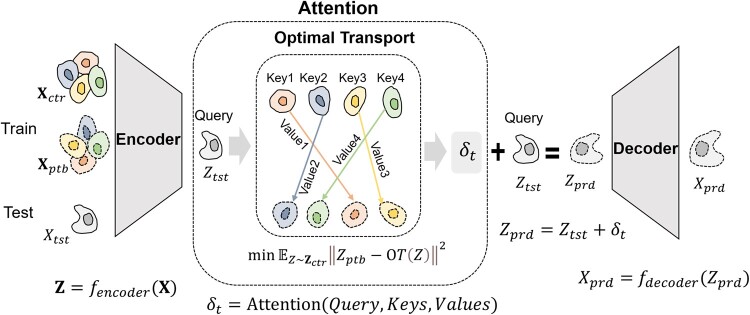
Overview of scPRAM. scPRAM employs the encoder of a VAE to map the cell-gene expression matrix into a latent space. Then, optimal transport is utilized to compute the coupling matrix between cells before and after perturbation, facilitating the matching of each unperturbed cell with its corresponding perturbed cell. Considering the latent vector of the test cell as a query, the latent space of the matched training set as a corpus, scPRAM employs attention mechanism to calculate the perturbation vector corresponding to each query. The perturbation vectors are added to their respective queries, and the predicted perturbation response is obtained through the output of the decoder.

The main framework of scPRAM consists of a variational autoencoder, optimal transport, and attention mechanism (Fig. 1). The VAE is primarily responsible for encoding the unpaired training set data of high dimensionality and sparsity into a latent space. Optimal transport is used to match cells before and after perturbations, which is very different from CellOT, which uses it directly for prediction. The attention mechanism is used to compute the perturbation vector for the test cell to be predicted.

For a given cell-gene expression matrix pair, i.e. $X_{\mathrm{ctr}}$ and $X_{\mathrm{ptb}}$, before and after perturbation, respectively, the well-trained VAE can encode them into lower-dimensional latent distributions, i.e.

Zctr=fencoder(Xctr)Zptb=fencoder(Xptb)

Due to the destructive nature of sequencing, cells before and after perturbation are not paired, and as a result, the data in $Z_{\mathrm{ctr}}$ and $Z_{\mathrm{ptb}}$ are not one-to-one correspondences. Here, the optimal transport (see Section 2.3) is employed to match cells before and after perturbation, thereby obtaining the optimal coupling matrix M between $Z_{\mathrm{ctr}}$ and $Z_{\mathrm{ptb}}$ (Flamary *et al.* 2021). By identifying the positions p where the maximum values in each row of M are located, we can match each cell before perturbation to a cell after perturbation, thus obtaining the adjusted $Z_{\mathrm{ptb}}$:

M=optimal transportZctr,Zptbpi=argmaxjMijZptb'=Zptb,p

After obtaining the paired cells, we can compute the corresponding perturbation vector $\delta_{i}$ for each cell pair, and all these perturbation vectors form the matrix Δ. Then, for each target cell to be predicted before perturbation, we encode its gene expression vector $x_{t}$ into a latent vector $z_{t}$ using the encoder of the VAE. Subsequently, scPRAM employs an attention mechanism (see Section 2.4) to calculate the corresponding perturbation vector $\delta_{t}$, which is then added to $z_{t}$. The output is decoded using the VAE decoder to obtain the predicted perturbation response. These processes can be represented by the following mathematical formula:

δi=Zptb,i'-Zctr,izt=fencoderxtδt=attentionzt, Zctr,Δxt,prd=fdecoderzt+δt

### 2.2 Variational autoencoder

Given the high dimensionality, sparsity, and cell-type specificity of cellular gene expression data, we employ a VAE to map the cell-gene expression matrix to a lower-dimensional latent space. The VAE architecture consists of an encoder $Q_{\varphi }\left(z|x\right)\;$mapping input x to a latent space distribution $q_{\varphi }\left(z|x\right)$, and a decoder $P_{\theta }\left(x|z\right)$ generating data samples x′ from latent variables z. To learn the latent space distribution, VAE employs variational inference to minimize the KL divergence between the approximate posterior $q_{\varphi }\left(z|x\right)$ and the true posterior $p_{\theta }\left(z|x\right)$:

KLqφzx ||pθzx=Ez∼qφzxlogqφzx-logpθzx

Using the Bayesian principle and formula transformation, the above expression can be rewritten as:

log⁡pθx-KLqφzx ||pθzx=Ez∼qφzxlogpθxz-KLqφzx ||pθz

The right-hand side of this equation represents the evidence lower bound (ELBO), which serves as a lower bound for the log-likelihood. At this point, maximizing the log-likelihood can be reformulated as maximizing the ELBO. From this, we can derive that the loss function for the VAE should be:

LossVAE=-Ez∼qφzxlogpθxz+αKLqφzx ||pθz

In this equation, $p_{\theta }(z)$ is typically set to the standard normal distribution N(0,1). To facilitate training, VAE employs the reparameterization trick. A sample z is obtained as z = μ + εσ, where μ and σ are encoder outputs and ε is noise from N(0,1). This enables us to efficiently update the parameters during training via backpropagation.

### 2.3 Optimal transport

The core idea of optimal transport is to find an optimal mapping between two probability distributions, minimizing the distance cost, to transform one distribution into another (Santambrogio 2015). In our research context, $Z_{\mathrm{ctr}}$ and $Z_{\mathrm{ptb}}$ represent the latent representations of gene expression matrices of cells before and after perturbation, with sample sizes of n and m, respectively. Due to the lack of prior knowledge, we assumed in the experiment that the distribution of cell samples before and after perturbation both follows a uniform distribution:

a=(1n, 1n, …, 1n)∈R1× n, b=(1m, 1m, …, 1m)∈R1× m

Then, we use the Euclidean distance to calculate the cost matrix C between the samples before and after perturbation:

C=Euclidean distance(Zctr,Zptb)∈Rn× m

Our goal is to find an optimal coupling matrix M that minimizes the total transportation cost. Therefore, the optimal transport problem is equivalent to the following optimization problem:

M=argminM⁡<M, C>Fs.t.M·1=aMT·1=bM≥0

in which $<M,\;{C>}_{F}$ denotes the Frobenius inner product. The minimum transportation cost in this context is also referred to as the Wasserstein distance (Panaretos and Zemel 2019), which is commonly used to measure the similarity between two distributions.

This problem can be solved using the Sinkhorn algorithm (Cuturi 2013), the main steps of which are as follows. Set the initial value of $v^{0}$ to 1, and then iterate over both sequences u and v until convergence:

ul+1=aKνl, vl+1=bKul+1

in which $K={\mathrm{e}}^{-\frac{C_{ij}}{\varepsilon }}.$ After iterative convergence, we obtain $u^{*}$ and $v^{*}$, and the approximate optimal coupling matrix M can be represented as:

M=Diagu*K Diagv*∈Rm× n

The element $m_{ij}$ in the *i*th row and *j*th column of this matrix denotes the proportion for transporting the *i*th unperturbed cell to the *j*th perturbed cell. We believe that paired cells have closer distances in latent space, which is reflected in larger transportation proportions in optimal transport. Therefore, for each row of the M matrix, we find the index of the column with the maximum value. This index represents the perturbed cell matched with the corresponding unperturbed cell.

### 2.4 Attention mechanism

For each cell under test, we consider using the attention mechanism to compute its perturbation vector. The attention mechanism allows the model to allocate different weights to different parts of the input data based on their importance, enabling more accurate predictions.

In our research context, the latent vector $z_{t}\;$of each cell under test serves as a query. The set of latent vectors from pre-perturbed cells in the training set serves as keys, and the corresponding perturbation vectors serve as values. For the query $z_{t}$, we first calculate its cosine similarity with each vector in $Z_{\mathrm{ctr}}$, obtaining the corresponding similarity coefficients ***s***:

si=zt⋅Zctr,iztZctr,i, i=1,2,…,n

Then, a certain proportion *β* is used to select the top $n_{t}$ coefficients with the highest similarities for Softmax normalization.

nt=βnit=argsort-s:ntp=softmaxsit=ⅇsit∑i=1ntⅇsit

Finally, we use a weighted sum approach to compute the perturbation vector δ for the cell under prediction:

δt=∑j=1ntpjδit,j

### 2.5 Data collection

We collected five datasets involving perturbations such as drug stimulation and bacterial infection. These datasets comprise different cell types, species, and individuals, making them suitable for studying predictions across various levels.

The PBMC dataset comprises human peripheral blood mononuclear cells (PBMC) stimulated with interferon-beta (IFN-β) and their control group. The original data can be accessed from the online Gene Expression Omnibus (GEO) database under the identifier GSE96583 (Kang *et al.* 2018). In this study, preprocessed data from scGen is utilized, encompassing seven distinct cell categories, totaling 18 868 individual cells.

The Hpoly.Day10 dataset includes intestinal epithelial cells infected with H.poly parasites for 10 days and control group. The original dataset is available on GEO under the identifier GSE92332 (Haber *et al.* 2017). In our study, processed data from scGen is employed, incorporating eight different cell categories, including 5951 cells totally.

The Nault dataset involves stimulating mouse liver cells with the carcinogen TCDD and control group. The original data can be accessed from the GEO database under the identifier GSE184506 (Nault *et al.* 2022). After Scanpy’s standard preprocessing pipeline (Wolf *et al.* 2018), the dataset comprises eight distinct cell types, totaling 29 030 individual cells.

The Species dataset consists of macrophage cells perturbed with lipopolysaccharide (LPS) for 6 h, along with their control group. This dataset is stored in the ArrayExpress database under accession number E-MTAB-6754 (Hagai *et al.* 2018). The dataset includes four different species: mice, rats, rabbits, and pigs. After Scanpy’s standard preprocessing pipeline, the dataset retains 15 528 individual cells.

The cross-individual dataset is derived from the Open Problems competition (Daniel 2023). The organizers provide PBMC data from three donors, encompassing five cell types subjected to 144 drugs. Detailed datasets can be downloaded from the corresponding Kaggle webpage: https://www.kaggle.com/competitions/open-problems-single-cell-perturbations/data.

## 3 Results

### 3.1 scPRAM can accurately predict perturbation responses in out-of-sample scenario

To demonstrate the accuracy of scPRAM in predicting out-of-sample scenarios, we conducted tests on the first four perturbation datasets. Although all four datasets contained annotations for cell types, we only used the cell type labels when splitting the training set. Specifically, we removed all the data perturbed for a certain cell type, leaving only the remaining data to predict the removed portion. Consequently, each dataset can be divided into several sub-experiments based on the number of cell types.

**Figure 2.**
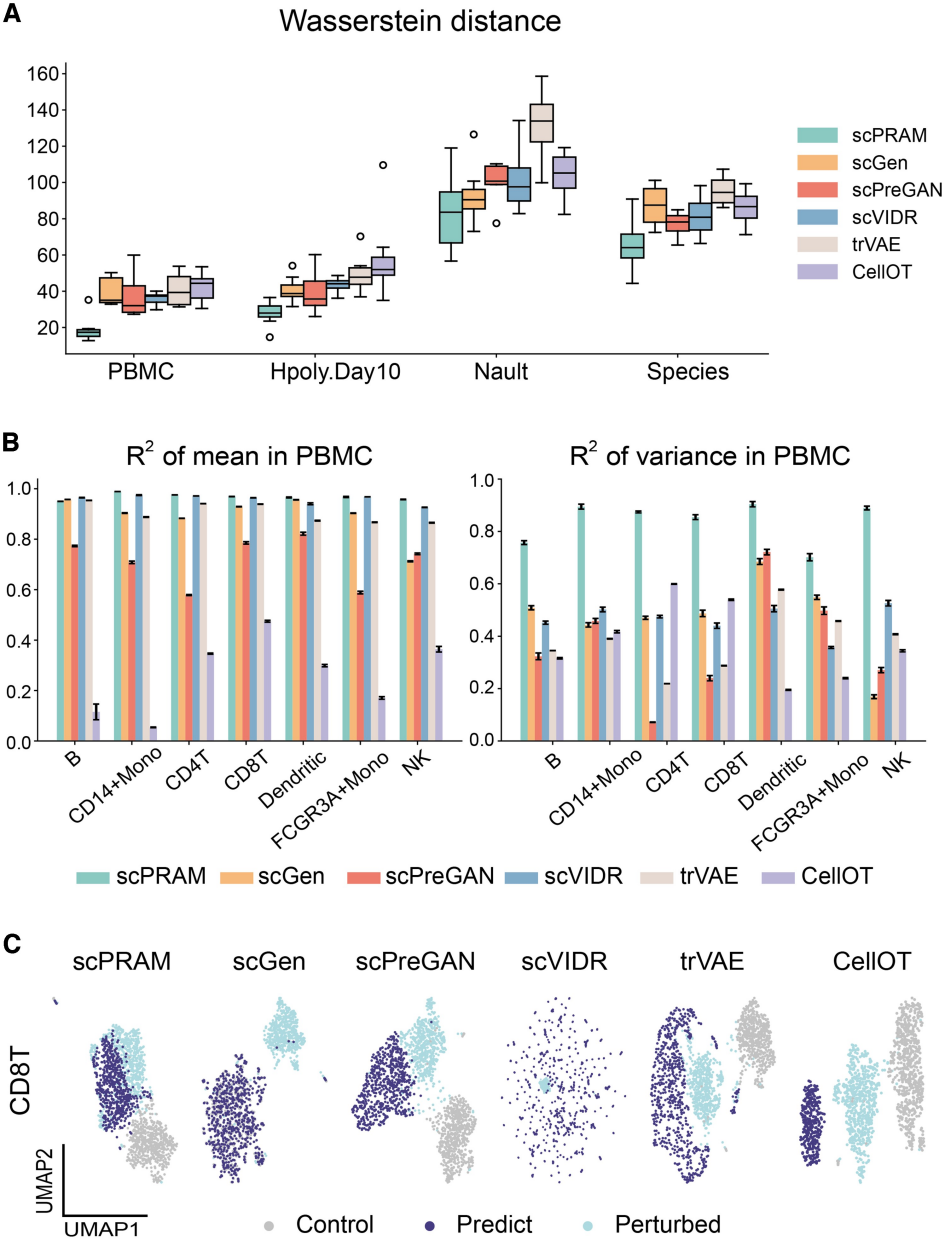
Evaluation of the accuracy of scPRAM in predicting perturbation responses. (A) Comparison of Wasserstein distances between predicted responses and actual responses for all the genes on the first four datasets. The data for each box are calculated from several sub-experiments within the corresponding dataset. (B) Determination coefficients for the mean and variance of gene expression in predicting responses versus actual responses for all genes in the PBMC dataset. Each bar chart is obtained by randomly sampling 80% of the cells, repeating this process 100 times, and calculating the corresponding mean and standard deviation. (C) UMAP visualization comparison of gene expression of CD8T cells from the PBMC dataset under different conditions.

First, we assessed the similarity between the predicted perturbation response and the actual perturbation response. Given that the samples before and after perturbation are not paired, direct calculation of metrics like mean squared error between $X_{\mathrm{prd}}$ and$\;X_{\mathrm{ptb}}$ is not feasible. Here, Wasserstein distance (see Section 2.3) is used to measure the distance between two data distributions and a smaller distance indicates higher similarity and more accurate predictions. In each of the four datasets, we calculated the Wasserstein distance between the predicted perturbation response and the actual perturbation response for the top 100 DEGs. It’s found that scPRAM-predicted perturbation responses had a greatly smaller Wasserstein distance to the actual perturbation responses compared to the other five methods across the four datasets (Fig. 2A).

Next, we performed regression on the mean and variance of gene expression between the predicted response and the actual response. Mean regression reflects the accuracy of predicting the overall expression status of the cell population under test, while variance regression takes into account the heterogeneity of single-cell gene expression. scGen and scVIDR have achieved good predictions on gene expression means but still have significant room for improvement in variance regression. The prediction effect of CellOT is mediocre, which is consistent with the results reproduced in scVIDR. In contrast, scPRAM not only predicts gene expression means accurately but also achieves a notable breakthrough in gene expression variance regression (Fig. 2B, Supplementary Fig. S1A).

To provide a more intuitive view of the accuracy of scPRAM predictions, we conducted UMAP (Becht *et al.* 2019) visualization on the gene expression results of CD8T cells from the PBMC dataset under different conditions. The UMAP visualizations of different methods show that the perturbation response predicted by scPRAM aligns very well with the actual response in low-dimensional space (Fig. 2C). In contrast, results predicted by the other four methods have significant bias. This demonstrates that scPRAM has a clear advantage in predicting perturbation responses (for more comparisons, see Supplementary Fig. S1B and C).

### 3.2 scPRAM can more accurately identify differentially expressed genes

**Figure 3.**
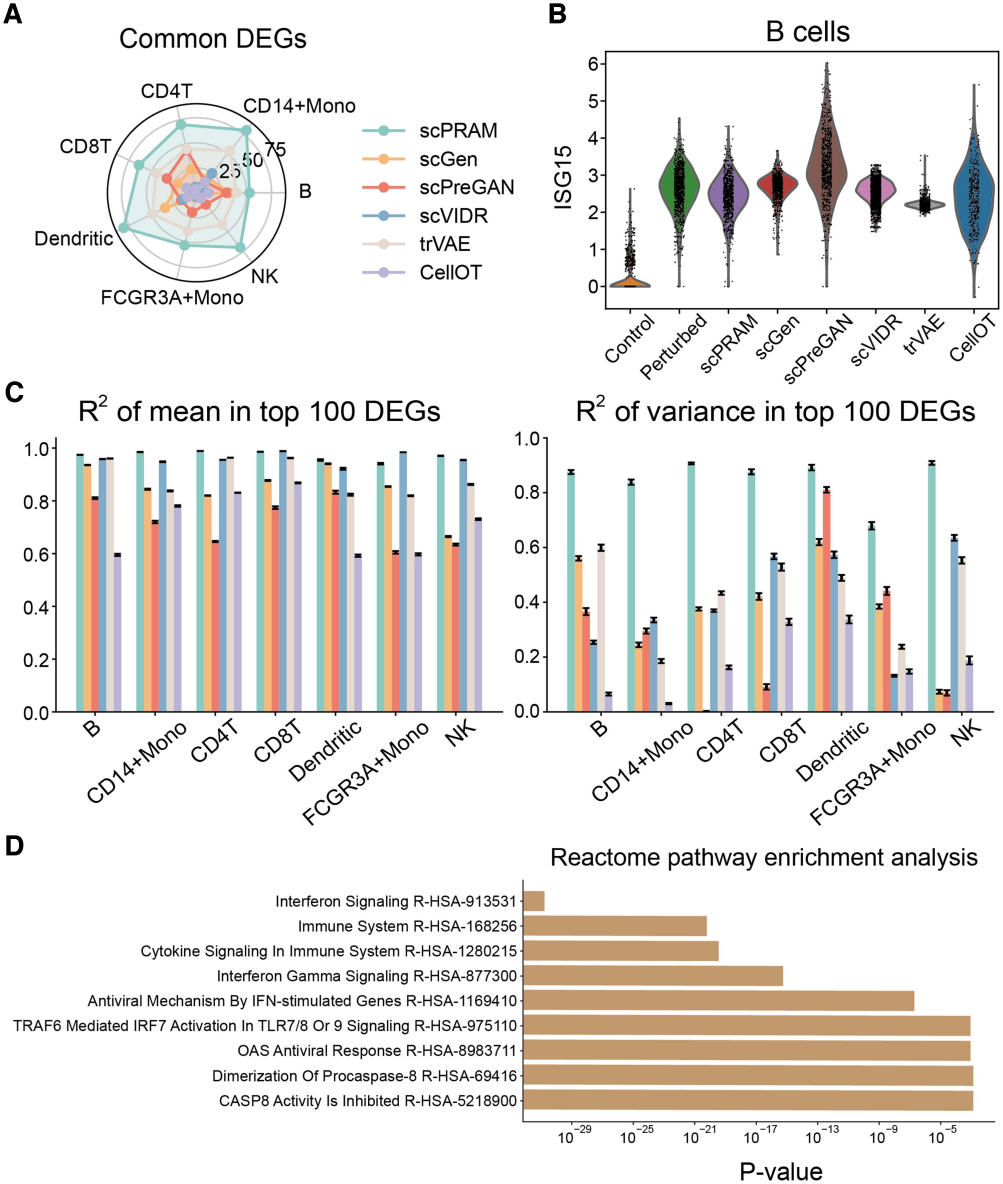
scPRAM accurately identifies and predicts DEGs. (A) Radar chart of the number of the top 100 common DEGs between true and predicted by different methods in each sub-experiment of PBMC dataset. (B) Violin plot comparing the gene expression of ISG15 in B cells of the PBMC dataset. (C) Regression scatterplots of mean and variance of gene expression for CD4T cells in the PBMC dataset, where each point represents a gene, and red points represent the top 10 DEGs of that type. (D) Highly correlated response pathways obtained from gene enrichment analysis using the top 100 predicted DEGs from the PBMC dataset with the Reactome 2022 gene database through the Enrichr website.

Accurately identifying DEGs allows for the analysis of perturbation-related pathways at the gene level, which is important for analyzing the impact of perturbations on other omics (Rapaport *et al.* 2013). Here, we used the Wilcoxon rank-sum test provided in Scanpy (Wolf *et al.* 2018) to calculate DEGs under different conditions. On the first four datasets, scPRAM identifies a significantly larger number of common DEGs, especially in PBMC and Hpoly.Day10 datasets, where scPRAM can identify nearly 50 or more DEGs. This result is much better than the other five methods (Fig. 3A, Supplementary Fig. S2A). To discern the advantages of scPRAM in predicting DEGs with greater precision, we have generated graphical representations of the top 20 DEGs obtained through various prediction methods for the PBMC dataset (Supplementary Fig. S2B).

In addition to identifying a larger number of DEGs, scPRAM can also predict the perturbation response of DEGs more accurately. To substantiate this point, we selected the top 100 DEGs from the PBMC dataset and conducted linear regression on their mean and variance. The results demonstrate a significant advantage of scPRAM, especially in the regression of variance on these top 100 DEGs (Fig. 3C). Furthermore, we depicted expression distribution plots for the ISG15 gene across various cell types, which reveal that the distribution predicted by scPRAM is in closer to the true perturbation distribution (Fig. 3B, Supplementary Fig. S2C).

Finally, we utilized the DEGs identified by scPRAM for gene enrichment analysis. Taking the PBMC dataset as an example, we inputted the top 100 DEGs identified by scPRAM into the online gene enrichment analysis tool Enrichr (Chen *et al.* 2013) and performed enrichment analysis against the Reactome 2022 (Gillespie *et al.* 2022) gene library (Fig. 3D). The enrichment results revealed that the top 100 DEGs predicted by scPRAM were most strongly associated with the interferon signaling pathway, aligning perfectly with the IFN-β perturbation observed in the PBMC dataset. Furthermore, examination of other highly correlated pathways indicated that IFN-β stimulation is closely linked to the immune system. Medically, it is well-established as a crucial immune regulatory protein primarily responsible for countering viral infections and modulating immune system activity (Zhang *et al.* 2015). This highlights the significant potential of scPRAM in identifying DEGs for downstream analysis, including the determination of response pathways.

### 3.3 scPRAM can better reveal the heterogeneity of cross-species perturbation

The research on cross-species perturbation prediction is of great significance because it helps in translating results from animal experiments to humans, which is crucial for clinical medicine. Predicting heterogeneous responses to the same perturbation across species remains a challenging problem due to the distinct response patterns in different species. Here, we illustrate this issue using the Species dataset, obtained through a 6-h stimulation of macrophages from four species including mice, rats, rabbits, and pigs with LPS (Supplementary Fig. S3A). On the data set partition, we iteratively hold out data from one species after perturbation and use the remaining data as the training set.

**Figure 4.**
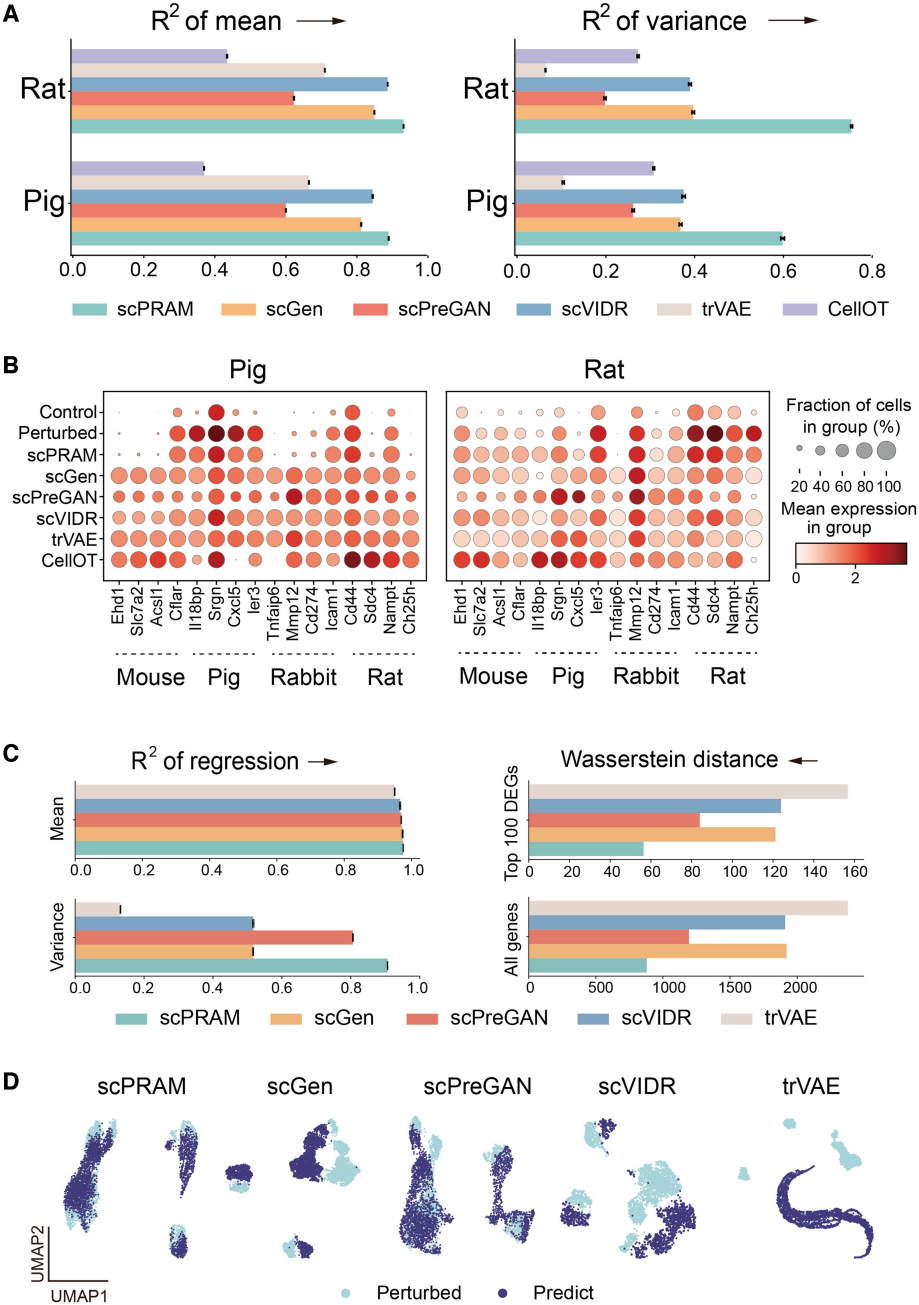
scPRAM can better reveal the perturbation heterogeneity among species and individuals. (A) Comparison chart of results from different methods for cross-species perturbation prediction. The left plot describes the regression coefficient of the mean expression of all genes between true perturbations and predicted perturbations, while the right plot describes the Wasserstein distance between true perturbations and predicted perturbations. (B) The expression states of 16 marker DEGs belonging to different species before perturbation, after perturbation, and in predictions from different methods. Two separate plots are generated using pig and rat data. Different genes correspond to the species below the horizontal lines. (C) The regression coefficients of gene expression mean and variance between predicted and true perturbation responses (left), as well as the Wasserstein distance between predicted and true perturbations for all genes and the top 100 DEGs (right). (D) UMAP visualization colored with different methods predicting the perturbation response of the third donor compared to the actual perturbation response.

First, we aim to investigate the overall accuracy of different methods in cross-species prediction of perturbation responses. In this case, the regression coefficient of the mean and variance value of gene expression were used as indicators for evaluation. The results in Fig. 4A indicate that there is substantial room for improvement in the cross-species prediction accuracy of existing methods, as their determination coefficients of variance of gene expression fall below 0.4. In contrast, scPRAM demonstrates superior performance in this regard, increasing the determination coefficients of variance of gene expression to over 0.6 and achieving the bigger determination coefficients of mean of gene expression.

More crucially, we investigated the heterogeneity of responses among different species. Initially, we selected the top four DEGs in each species that ranked in the top 20 for differential expression scores and did not appear in the top 20 of any other species, which were considered as marker DEGs for each respective species. Subsequently, we depicted the expression states of these genes in response to perturbation, both before and after the perturbation, as well as in the perturbation responses predicted by various methods across different species. The results in Fig. 4B revealed that scPRAM was better at capturing the heterogeneity in responses among different species to LPS stimulation. For instance, in the perturbation response of pigs, four marker DEGs belonging to mouse exhibited no pronounced response before and after perturbation. scPRAM effectively captured this phenomenon, whereas other methods predicted an upregulation in the expression levels of these genes. Besides, for four marker DEGs specific to pigs, scPRAM-predicted expression states were closer to the true responses compared to the predictions of other methods. Similar phenomena can be observed in other genes and across different species. This indicates that scPRAM is better at learning the heterogeneity of perturbation responses between species, which may be because other methods tend to rely on the average levels of existing species or the distribution of a certain species when predicting perturbation responses for new species, lacking exploration of the specific characteristics of the new species' input data. In contrast, scPRAM takes into account the similarities between data from the new species and data from existing species, making it better at identifying the appropriate perturbation directions.

### 3.4 scPRAM can predict perturbation responses in new patients more accurately

Due to inter-individual variability, different patients exhibit subtle differences in response to the same drug perturbation (Lauschke and Ingelman-Sundberg 2016). Using perturbation data from existing patients to predict perturbation responses in new patients can be beneficial for precision medicine. Here, we utilized the fifth dataset, comprising PBMC cells treated with belinostat from three donors, to investigate cross-individual perturbation response prediction (Supplementary Fig. S3B). We used data from the first two donors as a training set to predict the perturbation response of the third donor. After predicting this task with default parameters, CellOT yielded an almost zero determination coefficient; therefore, we did not compare it with our approach. Since there was no significant batch effect in the gene expression data of the three donors, all five methods achieved good performance in regressing the gene expression mean against the predicted perturbation response to the actual response, with regression coefficients close to 1. However, only scPRAM achieved *R*^2^ values close to 0.9 in variance regression. Similarly, for all genes and the top 100 DEGs, scPRAM had the smallest Wasserstein distance between predicted and true perturbation responses (Fig. 4C).

We also used UMAP to visualize low-dimensional manifolds of perturbation response predictions from different methods compared to true responses. Figure 4D shows that the results predicted by scPRAM align most closely with the true perturbation responses, while predictions of scGen and scVIDR are close but not entirely overlapping, likely due to biases in the data they generate. In this case, scPreGAN performs well, mainly because the individual differences are not significant, resembling an in-sample prediction scenario. However, its predictions ignore the original type information in the input data, leading to part data points lying between two types. In contrast, scPRAM retained the cell type information, even though the model did not utilize them during training.

### 3.5 scPRAM exhibits strong robustness

Considering that scRNA-seq data can be challenging to obtain in certain scenarios and often exhibits high levels of noise (Brennecke *et al.* 2013), we conducted a robustness analysis for different methods.

**Figure 5.**
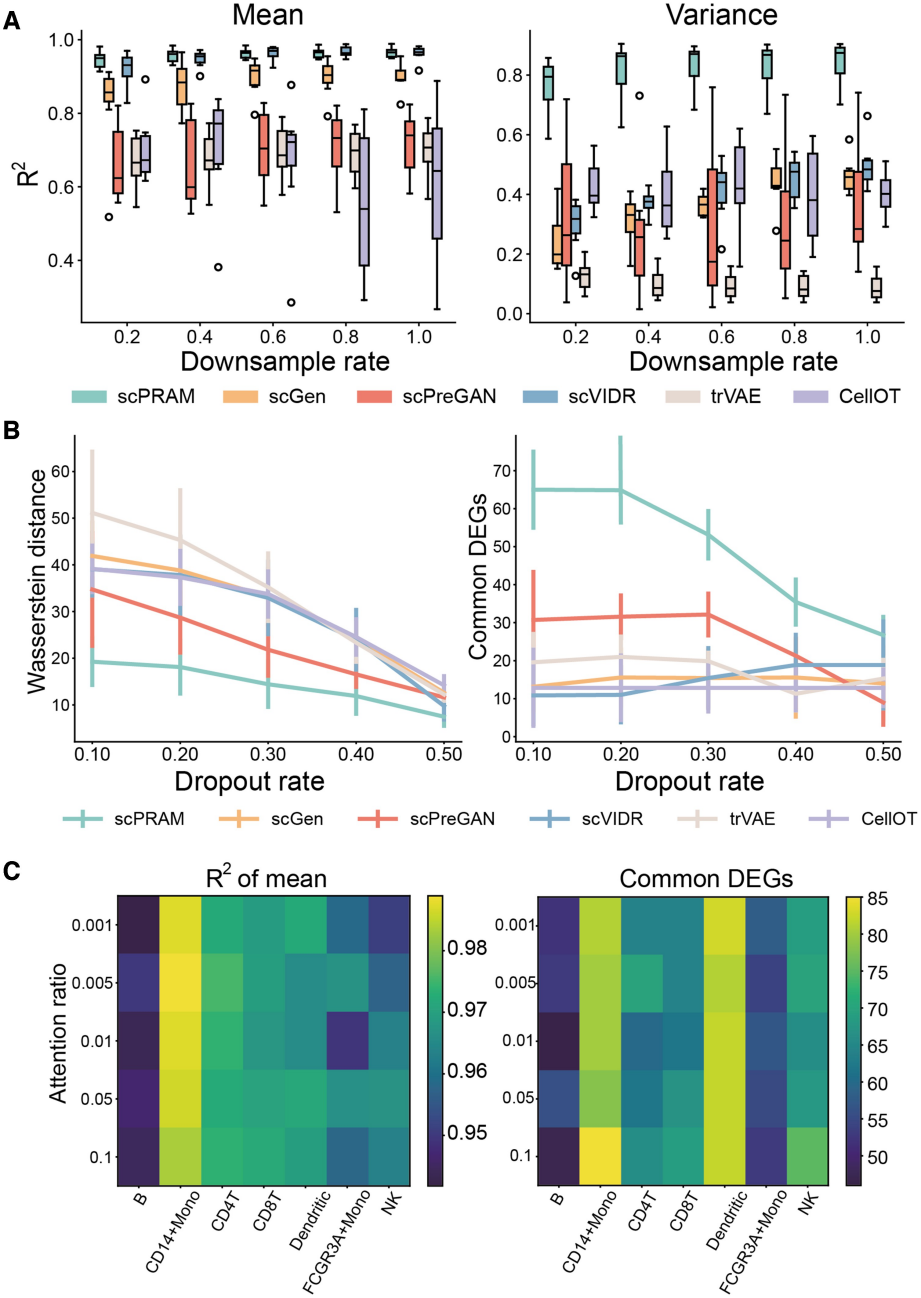
scPRAM exhibits strong robustness to experimental data and parameters. (A) The comparison of the results of predicting perturbation responses among five methods is presented under different sampling rates. Two boxplots represent the coefficients of determination of linear regression for the mean (left) and variance (right) of gene expression for all genes, with each point in the boxplot representing a cell type. (B) Line plots depicting the variation of Wasserstein distance (left) and the number of common DEGs (right) between perturbation responses predicted by different methods and the true responses, as the noise level varies. Each point is plotted using the mean and variance of seven sub-experiments of different cell types. (C) A heatmap showing the regression coefficient (left) of gene expression mean and the number of common DEGs (right) between the perturbation response predicted by different methods and the true response, as a function of the proportion of cells involved in the attention mechanism.

Here, taking the PBMC dataset as an example, we initially downsampled the dataset by sequentially sampling 20%, 40%, and up to 100% of the data from each cell type, with larger datasets encompassing the smaller ones. We then utilized regression coefficients of mean and variance of all gene expression to assess the performance of each method. It is evident that as the number of cells in the training set decreases, the performance of the baseline methods shows varying degrees of decline. However, scPRAM exhibits remarkable stability, with regression coefficients of gene expression mean consistently exceeding 0.9, and coefficients of gene expression variance hovering around 0.8. (Fig. 5A).

Next, we considered adding noise to the simulated data, where the approach involved randomly setting a proportion of the non-zero elements in the gene expression matrix to zero, with the proportion ranging from 10% to 50%. With increasing noise, the performance of all methods declined, but scPRAM consistently outperformed other methods under various noise levels (Fig. 5B). For imbalanced data, scPRAM also demonstrates excellent robustness (Supplementary Fig. S4).

Finally, we also consider the influence of hyperparameters of the scPRAM on the prediction performance. In the core attention mechanism algorithm, a key parameter is the proportion of training set cells used to calculate the perturbation vector. We varied the proportion parameter among 0.001, 0.005, 0.01, 0.05, and 0.1, and observed changes in gene expression mean and the number of common DEGs for each cell type. From Fig. 5C, it can be observed that the colors in the same column do not vary significantly. This suggests that scPRAM is not sensitive to the parameter of the proportion of cells involved in the attention mechanism.

## 4 Discussion

In this article, we introduce scPRAM, a single-cell gene expression perturbation response prediction method based on an attention mechanism. scPRAM uses a Sinkhorn-based optimal transport to match cells before and after perturbation in the latent space of VAE and employs an attention mechanism to compute perturbation vectors for new cells. Since OT automatically matches cells, scPRAM does not need to use type labels.

In scenarios involving out-of-sample prediction, scPRAM significantly outperforms existing state-of-the-art methods. Previous methods primarily focus on changes in the mean expression of a group of test cells, overlooking the distribution deviations among different cells after perturbation, which we assessed by using the variance of gene expression. scPRAM excels at identifying DEGs within perturbation responses. Through the identified DEGs, researchers can analyze perturbation response pathways and regulatory relationships at the gene level, better revealing the profound impacts of perturbations.scPRAM can be used not only to predict previously unseen cell types within the same cell lineage but also for cross-species and cross-individual predictions. In cross-species prediction experiments, scPRAM can better capture the heterogeneity of perturbation responses among species. In cross-individual predictions, scPRAM not only provides more accurate predictions of how new patients will respond to drugs but also effectively retains the biological information of the original cell categories.

While scPRAM has made significant progress in out-of-sample perturbation prediction, it, like most current methods, currently deals with single perturbations and has not yet considered perturbation covariates such as the drug dosage and the treatment duration. This is primarily because scPRAM focuses more on predicting responses to new types of samples under the same perturbation conditions. scPRAM has already demonstrated the superiority of the attention mechanism in perturbation response prediction, and exploring how to apply this strategy more broadly in more complex environments is a meaningful research topic.

## Supplementary Material

btae265_Supplementary_Data
