# Supplementary material for: scPRAM accurately predicts single-cell gene expression perturbation response based on attention mechanism
Source: Bioinformatics. 2024 Apr 15;40(5):btae265. doi: 10.1093/bioinformatics/btae265 (PMC11076148; doi:10.1093/bioinformatics/btae265)
Supplement: btae265_Supplementary_Data [file btae265_supplementary_data.docx]

**Supplementary Information for**

**scPRAM accurately predicts single-cell gene expression perturbation response based on attention mechanism**

Qun Jiang^1,ǂ^, Shengquan Chen^2,ǂ^, Xiaoyang Chen^1^, Rui Jiang^1*^

^1^MOE Key Laboratory of Bioinformatics and Bioinformatics Division of BNRIST, Department of Automation, Tsinghua University, Beijing, China

^2^School of Mathematical Sciences and LPMC, Nankai University, Tianjin, China.

**Contents**

[Supplementary Figures 2](#_Toc161154754)

[Supplementary Fig. S1 2](#_Toc161154755)

[Supplementary Fig. S2 3](#_Toc161154756)

[Supplementary Fig. S3 4](#_Toc161154757)

[Supplementary Fig. S4 5](#_Toc161154758)

# Supplementary Figures

## Supplementary Fig. S1


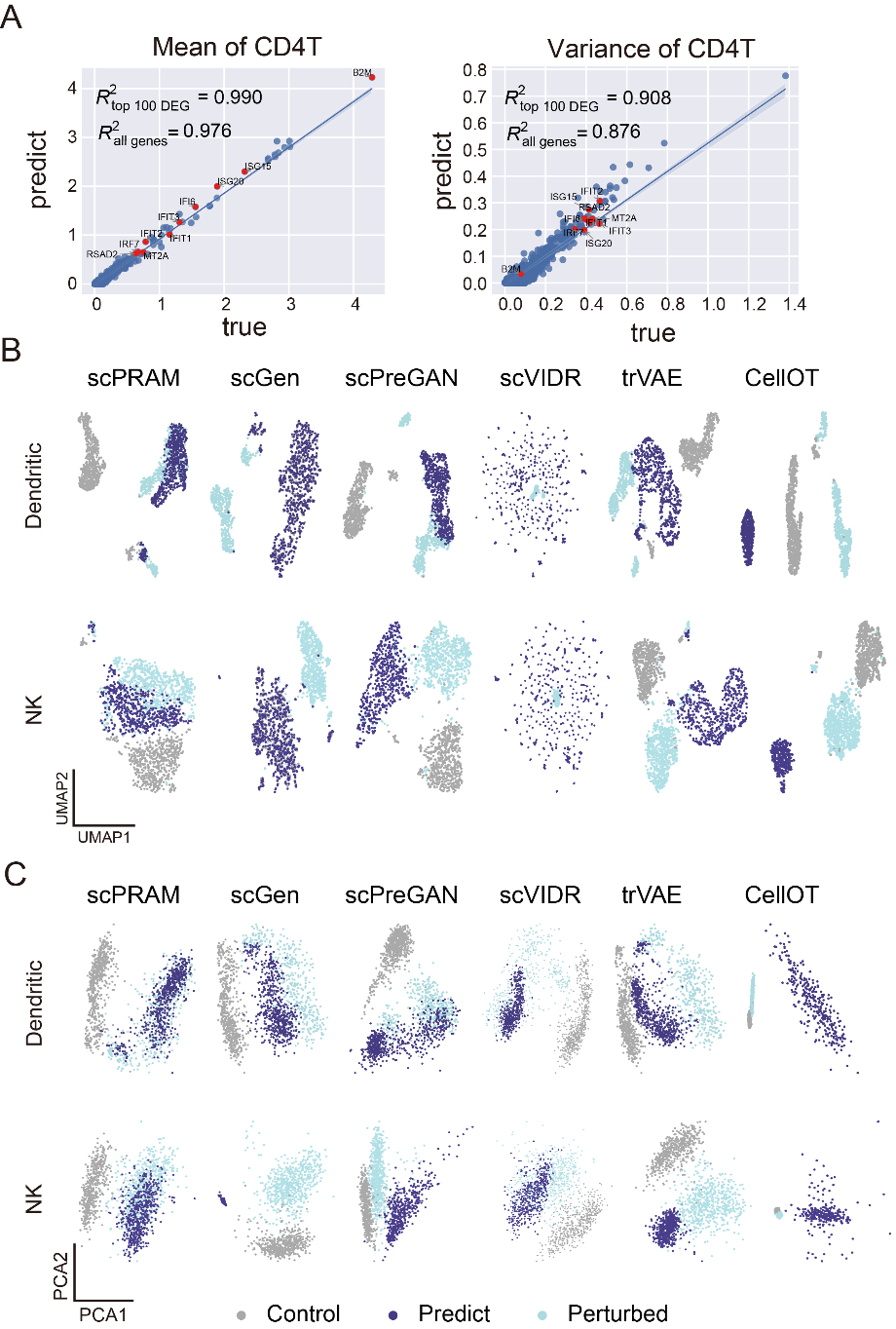


**Supplementary Fig. S1. More experimental results demonstrate that scPRAM predicts perturbation responses better than existing methods.** (A) Regression scatterplots of mean and variance of gene expression for CD4T cells in the PBMC dataset, where each point represents a gene, and red points represent the top 10 DEGs of that type. (B) UMAP visualization comparison of gene expression of dendritic and NK cells from the PBMC dataset under different conditions. (C) PCA visualization comparison of gene expression of dendritic and NK cells from the PBMC dataset under different conditions.

## Supplementary Fig. S2


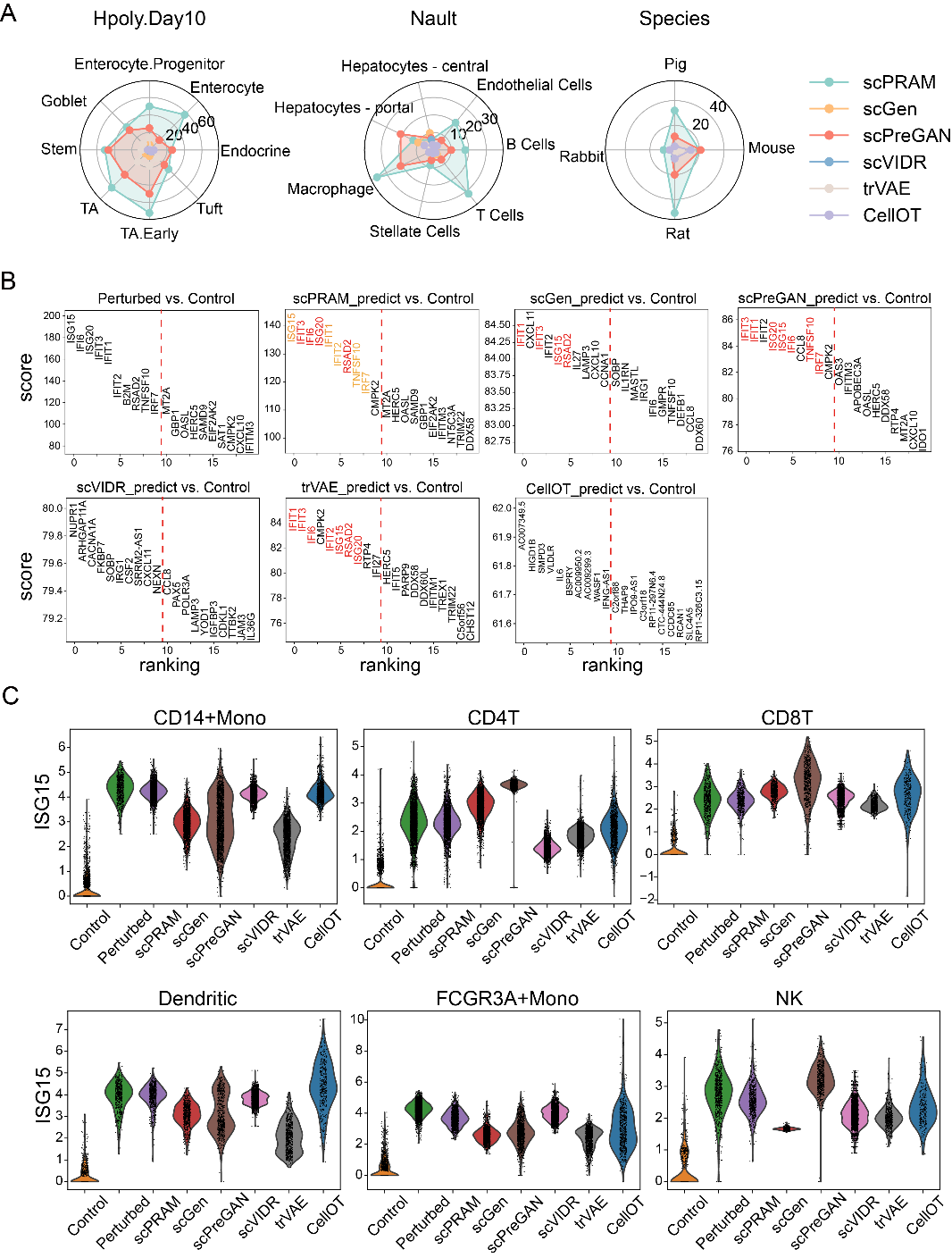


**Supplementary Fig. S2**. **More experimental results of scPRAM accurately identifies of differentially expressed genes.** (A) Radar chart of the number of the top 100 common DEGs between true and predicted by different methods in each sub-experiment across the three datasets. (B) The top 20 DEGs between the true perturbation and those predicted by five different methods and the control group. Among the top 10 DEGs, those highlighted in red correspond to the true DEGs, while those highlighted in gold share the same ranking. (C) Violin plot comparing the gene expression of ISG15 in six types cells of the PBMC dataset.

## Supplementary Fig. S3


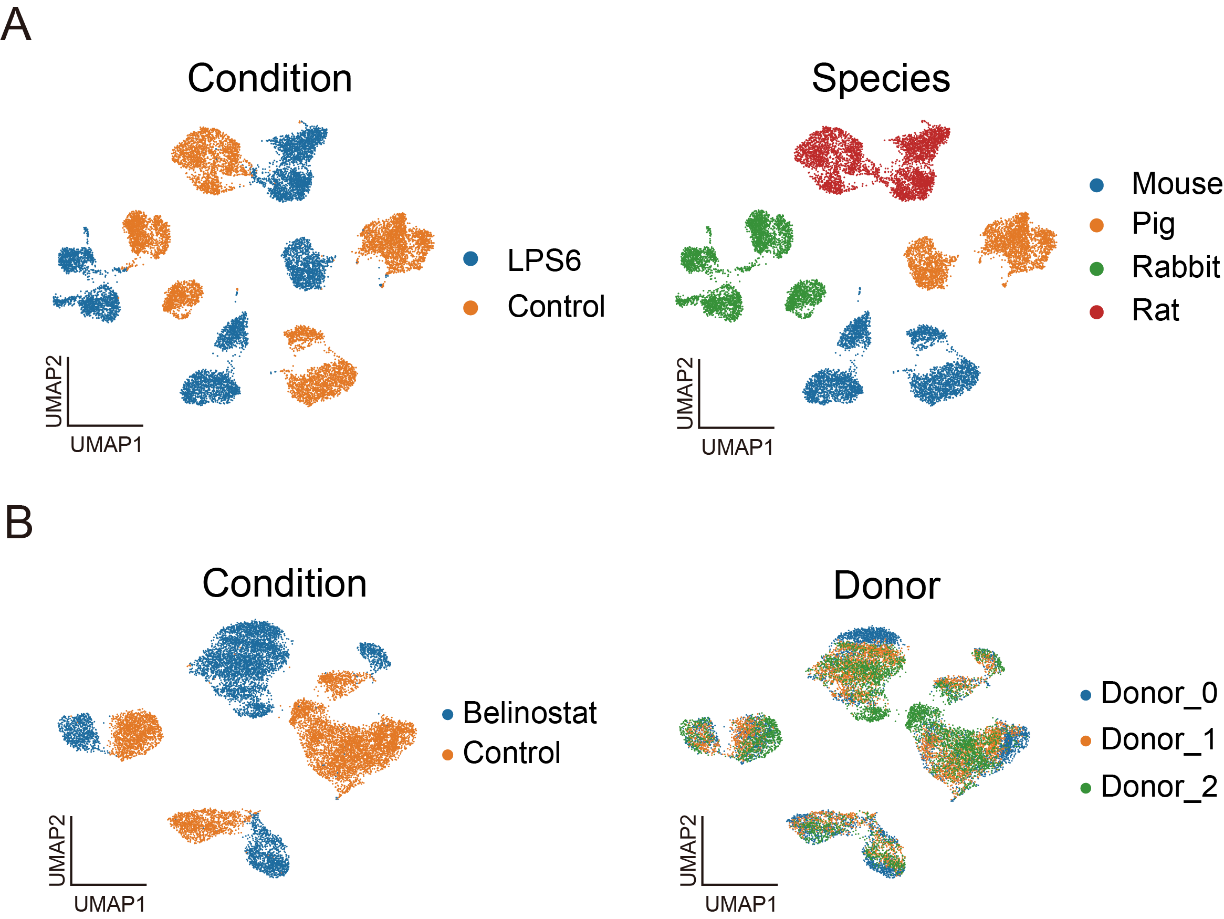


**Supplementary Fig. S3. UMAP visualization of data sets from cross-species and cross-individual experiments.** (A) UMAP visualization colored by condition (up) and species (down) for cross-species perturbation dataset. (B) UMAP visualization of PBMC gene expression data from three donors colored by condition and donor.

## Supplementary Fig. S4


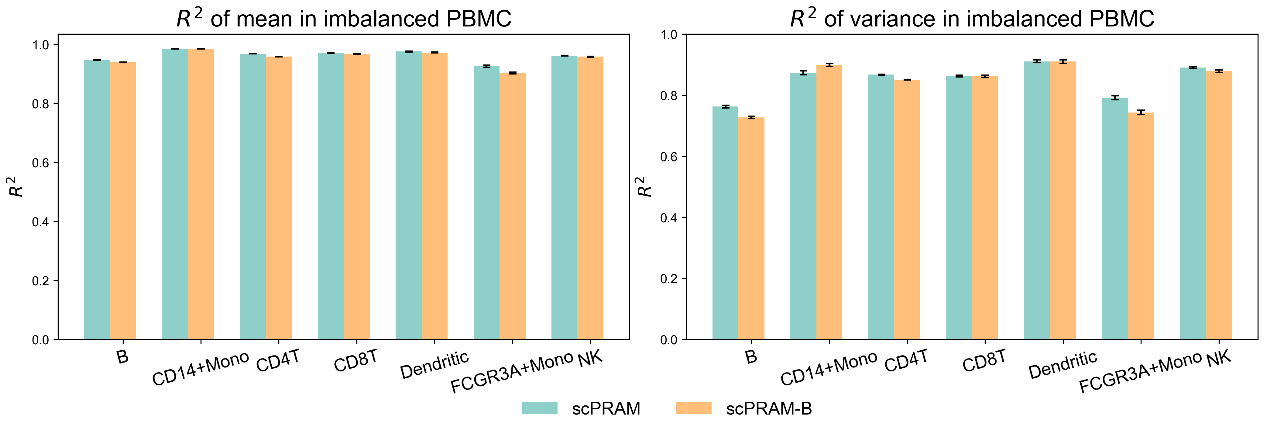


**Supplementary Fig. S4. The coefficient of determination for the linear regression of the mean and variance between the actual response and predicted response in imbalanced PBMC dataset.** scPRAM directly matches the imbalanced dataset and then predicts perturbation response. scPRAM-B balances the number of cells before and after perturbation through resampling before using the attention mechanism.
